# Novel semi-decentralised mobile system for the sanitization and dehydration of septic sludge: a pilot-scale evaluation in the Jordan Valley

**DOI:** 10.1007/s11356-021-17018-z

**Published:** 2021-10-31

**Authors:** Eva Kocbek, Hector A. Garcia, Christine M. Hooijmans, Ivan Mijatović, Mohammad Al-Addous, Zakariya Dalala, Damir Brdjanovic

**Affiliations:** 1 Department of Biotechnology, Delft University of Technology, Van der Maasweg 9, 2629 HZ Delft, The Netherlands; 2 Department of Environmental Engineering and Water Technology, IHE-Delft Institute for Water Education, Westvest 7, 2611 AX Delft, The Netherlands; 3 Tehnobiro d.o.o., Heroja Nandeta 37, 2000 Maribor, Slovenia; 4 Department of Energy Engineering, German Jordanian University, P.O.Box 35247, Amman, 11180 Jordan

**Keywords:** Mechanical dewatering unit, Faecal sludge, Microwave irradiation, Membrane separation technology, Nutrients, Resource recovery, Reuse

## Abstract

The provision of effective sanitation strategies has a significant impact on public health. However, the treatment of septic sludge still presents some challenges worldwide. Consequently, innovative technologies capable of an effective and efficient sludge treatment, mostly at a decentralized level, are in high demand to improve sanitation provision. To address this problem, this study evaluates a novel semi-decentralised mobile faecal sludge treatment system, the pilot-system for which consists of a combination of several individual processes including mechanical dewatering (MD), microwave (MW) drying, and membrane filtration (ultrafiltration [UF] and reverse osmosis [RO]). The system evaluation was carried out by treating raw, partially digested faecal sludge (FS) from septic tanks—hence, septic sludge (SS)—in the Jordan Valley, Jordan. The pilot-scale system exhibited an effective and flexible treatment performance for (i) sanitizing faecal sludge and related liquid streams (MW and UF); (ii) reducing the treated sludge mass (and sludge volume) (MD and MW); and (iii) producing a high-quality treated liquid stream ideal for water reclamation applications (UF and RO). The MD process removed approximately 99% of the initial SS water content. The MW drying system completely removed *E. coli* and dehydrated the dewatered sludge at low energy expenditures of 0.75 MJ kg^−1^ and 5.5 MJ kg^−1^, respectively. Such energy expenditures can be further reduced by approximately 40% by recovering energy in the condensate and burning the dried sludge, which can then be reused inland applications. The membrane filtration system (UF and RO) was able to produce high-quality treated water that is ideal for the water reuse applications that irrigation requires, as well as meeting the Jordanian standard 893/2006. In addition, the system can also be powered by renewable energy sources, such as photovoltaic energy. Therefore, this research demonstrates that the evaluated semi-decentralised mobile system is technically feasible for the in situ treatment of SS (sanitization and dehydration), while also being effective for simultaneously recovering valuable resources, such as energy, water, and nutrients.

## Introduction

Implementing effective strategies for the prevention of contagious and potentially deadly diseases outbreaks is a priority for sanitation providers worldwide. Faecal sludge (FS) contains various types of pathogenic organisms such as bacteria, viruses, and parasites; meaning that FS needs to be properly treated and disposed of in an environmentally sound manner (Singh et al. 2017). The proper collection, treatment, and disposal of FS have contributed significantly to the strengthening of public health, particularly, for those living under low-income conditions (Sykes and Skinner 2015). Pit latrines and septic tanks are the most common alternatives worldwide for FS collection (Connor et al. 2017; Thye et al. 2011). However, such collection facilities only enable the separation of faeces from human contact, so providing marginal treatment, thus, if not properly treated at a later stage, such large amounts of FS only accumulate, creating a subsequent waste disposal problem.

FS originating from on-site sanitation facilities, such as pits, vaults, and septic tanks, has an appearance of slurry or solid of fresh, undigested, or partially digested faeces with or without urine, water, and sanitary products (Tayler 2018). FS composition is highly variable depending on the design, construction, operation, and maintenance of the on-site sanitation facility where the sludge is generated and collected. The dry solid (DS) concentrations lower than 5% are commonly reported for FS from septic tanks or from poorly draining leach pits (Tayler 2018). Higher DS content have been reported in dry pit latrines compared to septic tanks or poorly draining leach pits, unless the water table in that particular region under evaluation is higher than the water level in the pit latrines and/or the flushing water from the toilets is discharged into the pit (Tayler 2018). Moreover, the sludge emptying frequency, temperature, rainfall patterns, and groundwater intrusion can all further influence the FS composition. In all instances, FS exerts a high variation in oxygen demand and solids content. For instance, FS from septic tanks, septic sludge (SS), may exhibit dry solid (DS) concentrations ranging from 0.5 to 121.6 g L^−1^, and chemical oxygen demand (COD) from 0.4 to 91.9 g L^−1^ (Englund et al. 2020; Gold et al. 2018; Strande et al. 2018). Similarly, large fluctuations in FS composition derived from pit latrines have been reported, with DS and COD concentrations ranging from 3.5 to 127.6 g L^−1^ and from 1.8 to 100 g L^−1^, respectively (Englund et al. 2020). The FS composition will determine either the final sludge disposal or the possibilities for implementing resource recovery strategies. Sludge resource recovery activities can include the reuse of the sludge in agricultural applications, and the recovery of energy via co-combustion, among others (Kacprzak et al. 2017).

On-site sanitation facilities are either manually or mechanically desludged (Singh et al. 2021). Once collected, these large amounts of FS need to be transferred either to disposal sites, or to treatment facilities usually located away from the points of generation, thus, increasing the overall disposal costs due to transportation expenditure (Mawioo et al. 2016a). Different alternatives are available for FS treatment, which include drying, composting, co-composting with organic solid waste, co-treatment with municipal sewage in wastewater treatment plants, and anaerobic digestion with or without organic solid waste, among others (Ronteltap et al. 2014). For example, when treating FS in sludge drying beds, the sludge moisture is reduced through percolation/filtration and evaporation processes (Bui et al. 2019; Kengne et al. 2019; Moiambo et al. 2021; Mrimi et al. 2020; Nikiema and Cofie 2014). The essential advantage offered by sludge drying beds is that such systems do not require electrical energy to operate; i.e., no energy is needed for the dewatering/drying of the sludge (Nikiema and Cofie 2014). In such scenarios, more than half of the faecal sludge management costs can be attributed to sludge transportation costs (Murungi and van Dijk 2014; Steiner et al. 2002). Therefore, the sludge transportation costs component may exceed by far the sludge treatment costs. Thus, to tackle such high transportation costs, the scientific community has been researching technologies that enable the in situ sludge treatment (Forbis-Stokes et al. 2021; Septien et al. 2018). Furthermore, the application of the aforementioned FS treatment alternatives is usually limited by the low throughput treatment capacity of such systems that, as a result, present large footprint requirements. The implementation of such processes is, therefore, a major challenge in areas with a pattern of rapid FS generation and limited land availability. These scenarios are frequently encountered in densely populated areas, such as congested cities, slums, and emergency situations. So the challenge lies in finding solutions to manage FS in areas that require responsive sludge collection, transport, and treatment (Mawioo et al. 2017). As such, there is a need for more systematic, innovative, and versatile methodologies for the proper treatment and disposal of FS.

Mobilized semi-decentralized (MSD) technologies for FS treatment have received increased attention due to the flexibility that such a concept offers to both sludge treatment and management (Forbis-Stokes et al. 2021). MSD systems can be tailor-made to the needs of users, as well as being highly suited to both short- and long-term applications due to their robustness and cost-effectiveness. Furthermore, compared to centralized sludge treatment systems, MSD technologies offer additional advantages that include the in situ sludge treatment capacities, the ease of deployment, and the low investment and operational costs required, among others. In recent years, some MSD technologies have been developed for FS treatment; however, in terms of full-scale scenarios, such developments have been narrowly applied (Forbis-Stokes et al. 2021; Septien et al. 2018). Most likely, a combination of several technologies may be required for the provision of an effective and efficient treatment alternative.

Microwaves (MWs) are capable of converting electromagnetic energy into thermal energy. Consequently, the utilization of MW technology can serve to effectively dehydrate and reduce/eliminate the pathogen content of various types of sludge including blackwater sludge, SS, fresh FS from latrines, and sewer municipal sludge reaching DS concentrations of up to 90%. In addition, the inactivation of several pathogens has been demonstrated, which include *E. coli*, *Ascaris lumbricoides eggs*, *Staphylococcus aureus*, and *Enterococcus faecalis* (Hong et al. 2004; Hong et al. 2006; Mawioo et al. 2017; Mawioo et al. 2016a, b; Pino‐Jelcic et al. 2006). During MW heating, the substance being irradiated is penetrated by the MWs, so the heat generation and propagation occurs from the center of the material outward, hence, making for a more efficient heating process (Kumar et al. 2016; Stuerga 2006). In addition, MW systems can be immediately started up and shut down (without pre-heating the system before drying or cooling down after drying), which minimizes energy losses to the environment.

The task of thermal sanitization and drying demands rather a lot of energy depending on the initial amount of water present in the sludge (Léonard et al. 2004; Mawioo et al. 2017). As such, the use of a low-energy-demanding preliminary sludge dewatering process (mechanical dewatering [MD]) before applying the more energy-demanding drying processes (MW heating) can result in an overall cost-effective alternative. Such a strategy has been performed and proven quite effective at both centralized and decentralized wastewater treatment facilities (Nikiema and Cofie 2014; Schaum and Lux 2010). MD dewatering processes separate the water from the sludge with the use of screens, screws, belt presses, pressure filters, centrifuges, and vacuum filters, among others; thereby, increasing the DS content at relatively low capital and operational cost. The resulting sludge after MD may exhibit a DS content of up to approximately 30%. This also leads to a considerable volume reduction of the dewatered sludge by approximately 90% (Nikiema and Cofie 2014; Schaum and Lux 2010).

The water extracted from the sludge both by the MD processes and also the MW drying processes can be recovered for subsequent water reclamation applications. Such liquid streams contain (in addition to water) other valuable resources such as nutrients. However, they may also contain pathogenic organisms; in particular, the water rejected from the MD processes. Such streams must be treated, therefore, before valuable resources are recovered, as this will prevent health risks or environmental pollution. Direct membrane UF using ceramic membranes has been evaluated for the direct treatment of raw municipal wastewater and greywater, showing good performance in terms of the quality of water produced (permeate) (Das et al. 2018; Kramer et al. 2015, 2020). The permeate obtained from the direct ceramic membrane UF can be further treated by a reverse osmosis (RO) process. It is possible, therefore, to obtain a superior water quality free of suspended solids, bacteria, and viruses, at the same time as rejecting soluble compounds like ammonium (NH_4_^+^), and orthophosphate (PO_4_^3−^). The nutrients can be recovered in the concentrate of the RO membrane filtration for subsequent reuse for example, as crop fertilizers. As such, the application of UF in conjunction with RO pressure-driven membranes is highly recommended for treating the water streams and also for recovering resources (Hube et al. 2020; Kramer et al. 2015). This strategy is particularly relevant for regions facing water shortages, such as for Middle Eastern countries, as the recovered water (and other resources) represents a valuable water source for irrigation applications.

The use of specific treatment technologies for sludge treatment—such as MW treatment, MD systems, and membrane separation processes—has been individually demonstrated in specific applications; in particular, in the framework of centralized municipal wastewater treatment plants (Bennamoun et al. 2016; Chen et al. 2014; Das et al. 2018; Kramer et al. 2015, 2020; Mawioo et al. 2016b). However, the combination of such treatment technologies to aim for the development of an improved decentralized FS treatment alternative has not been yet carried out. In addition, the validation of such a combination of technologies in real-life scenarios is still pending (Forbis-Stokes et al. 2021; Nikiema and Cofie 2014; Septien et al. 2018). The proposed concept (train of technologies) is envisioned to be very energy demanding (particularly the MW drying units) and this could be challenging in low-income countries and/or in humanitarian settings. However, there are alternatives beyond powering the system by connecting to the local grid. Such alternatives include the provision of diesel generators, as well as powering the system using renewable sources such as solar/PV energy.

The present study is aimed at investigating the feasibility of a novel pilot-scale MSD unit for the in situ treatment of SS (drying, pathogen inactivation, and liquid streams treatment). The novel pilot-scale MSD unit included an MD unit, an MW drying system, and a membrane filtration system (UF and RO), while the feasibility of the evaluated technologies for resources recovery was also explored. The sludge treatment performance was assessed, first by characterizing the treated sludge and generated liquid streams properties, and, second, by determining the treatment performance of each individual set of technologies conforming to the pilot-scale system. Following this, the validation of the proposed technology was carried out in the Jordan Valley area by treating locally generated SS.

## Materials and methods

### Experimental unit

**Fig. 1:**
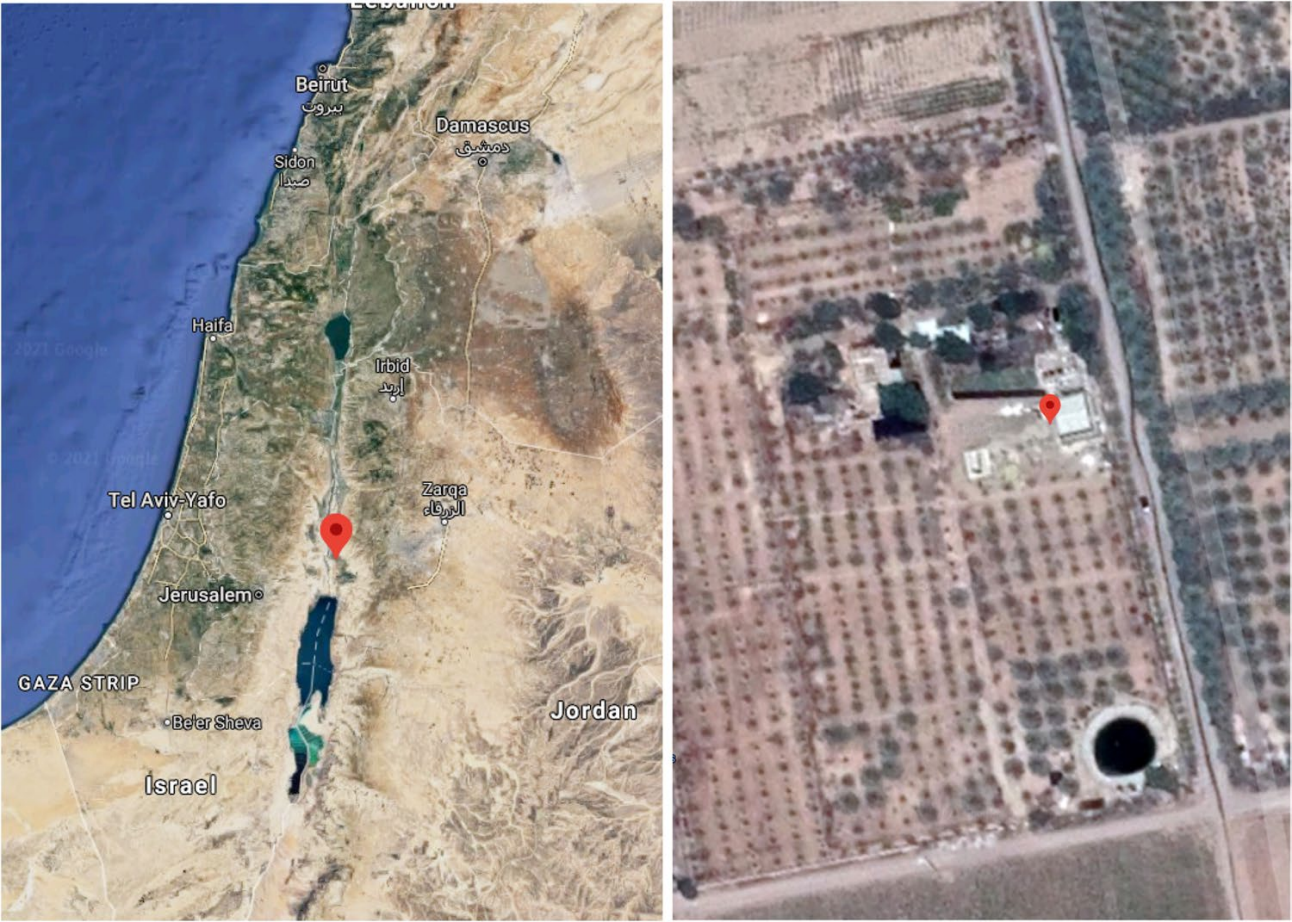
Project location at the Jordan Valley —geographical coordinates 31° 54′ 38.78″ N and 35° 34′ 40.63″ E ( source: Google Maps)

**Fig. 2:**
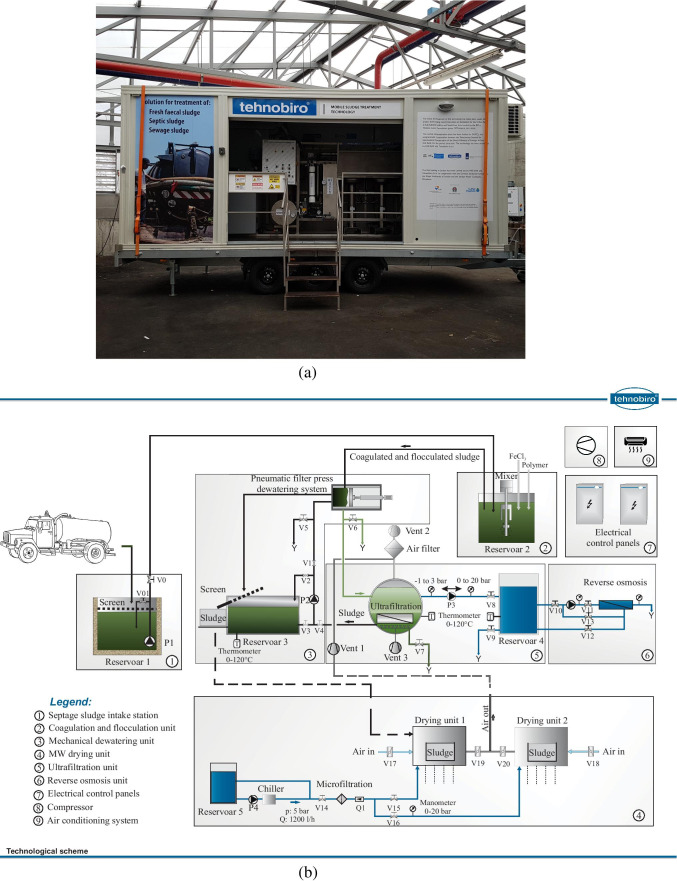
**a** General view of the pilot system; and **b** schematic representation (Tehnobiro d.o.o)

The MSD pilot-scale unit was designed and manufactured by Tehnobiro d.o.o (Maribor, Slovenia). The system performance was evaluated at the experimental facilities of the German Jordanian University (GJU) in the Jordan Valley area, Jordan (Fig. 1), with a focus on treating SS. The pilot system consisted of a combination of three key components: an MD unit, an MW drying system, and a membrane filtration (UF and RO) system. The system components were designed to be operated in a batch mode, allowing for robust process control and flexibility. The pilot system was built in a standard (six-meter length) cargo-size container and mounted on a trailer to facilitate the unit’s mobility. The pilot system was designed for the treatment of approximately 130 L h^−1^ of SS from an initial DS concentration of approximately 2% to a final DS concentration of 85%. The evaluation of the pilot system was carried out in the winter season and the key prototype components are displayed in Fig. 2. A detailed description of all the system components is presented in the “Septage sludge intake station and coagulation and flocculation unit” section to the “Membrane separation system” section.

#### Septage sludge intake station and coagulation and flocculation unit

The SS intake station (#1 in Fig. 2) consisted of a 500-L polyethylene receiving tank, a rough screen, and a submersible pump with a floating level switch. The coagulation and flocculation unit (#2 in Fig. 2) consisted of a reservoir and a mixer placed immediately after the intake station and before the MD unit. The coagulation agent (ferric chloride) and flocculation agent (polymer (Drewlock™ 469)) based on acrylamide and a cationic co-monomer (Solenis, USA) were manually added into the unit to promote particle destabilization and flocculation of the suspended solids in the SS. In such a way, larger particles were formed that could be better separated in the downstream MD unit. The coagulation and flocculation methods are described in the “Experimental procedure” section.

#### Mechanical dewatering unit

The MD unit (#3 Fig. 2) consisted of a 500-L equalization tank (500 × 1,000 × l,000 mm) equipped with a rough screen (30 mm), a water level transmitter, a pneumatic filter press dewatering system, and a centrifugal pump with an open impeller. The pneumatic filter press dewatering system consisted of a cylindrical stainless-steel chamber (125 mm diameter and 300 mm length) and was designed for a throughput capacity of 50 L h^−1^ (Tehnobiro d.o.o., Slovenia). Inside that cylindrical chamber, a 0.5 mm slotted cylindrical screen (filter) was placed. A stainless-steel piston pneumatically (operated by means of a manual hand lever valve) pushed the sludge through the cylindrical screen, which dewatered the sludge, while compressed air was supplied to the pneumatic unit by a compressor (#8 Fig. 2). The sludge dewatering unit produced two outputs: (i) the concentrated solid sludge cake and (ii) the liquid filtrate. The concentrated sludge cake was manually collected and further treated (dried) at the MW drying unit (“MW drying unit” section). The filtrate was discharged by gravity to the UF membrane system for further treatment (“Membrane separation system” section). As indicated in Fig. 2, the MD unit was also provided with a centrifugal pump for bringing the concentrate from the downstream membrane filtration process back to the MD unit. The concentrate could either be brought back to the MD unit for further dewatering or else processed (dried) directly in the MW system. The higher the DS concentration in the UF concentrate, the higher the opportunities to treat the UF concentrate directly in the MW drying unit. The fouling properties of the UF concentrate determined the maximum achievable DS concentrations in the UF concentrate, while the UF membrane fouling was monitored by measuring the pressure drop across the membrane.

#### MW drying unit

The MW drying unit (#4 Fig. 2) was designed to sanitize and dry the MD sludge by using MW radiation. The drying unit consisted of two cylindrical MW cavities, rotating polypropylene (PP) turntables, sludge PP holding vessels, a ventilation unit for vapour extraction, an air filtration system, two MW power supply sources, and two MW magnetrons with a power output capacity of up to 6 kW each, operated at a frequency of 2,450 GHz. In the present study, the MW unit was operated at a power output of 1.5 kW. The cavities were made of stainless steel with a diameter of 400 mm and a total length of 400 mm. The PP turntables were used to gently and continuously rotate the irradiated sludge at a speed of one rpm, which alleviated the effect of potential non-uniform temperature distributions. The PP holding vessels were designed for a maximum load of 6 kg each. The exhaust vapour extracted by the ventilation system was treated by the air filtration system composed of (i) activated carbon soaked in phosphoric acid, (ii) activated carbon soaked in sodium hydroxide, and (iii) aluminium oxide with potassium permanganate. The MW magnetrons and power supply sources were cooled down by recirculating demineralised water at a flow rate of 1,200 L h^−1^ and a 5-bar pressure. The changes in the moisture content of the sludge during MW drying were continuously measured by a single-point load cell (Mettler Toledo), while the temperature of the sludge was measured intermittently during the sanitization process using a thermal camera (FLIR TG 165, FLIR Systems Inc., USA) and a temperature gauge (ELPRO Lepenik & Co., Slovenia).

#### Membrane separation system

The membrane filtration treatment system is presented in #5 and #6 (Fig. 2). The sludge filtrates from the MD, together with the condensate produced from the evaporated water from the MW drying unit, were collected in the UF reservoir prior to the UF process. Within this reservoir, a ceramic UF membrane element with an average pore size of 0.08 μm and a total filtration area of 1.05 m^2^ was immersed and operated in a dead-end filtration mode for permeate extraction. An air diffuser manifold was installed at the bottom of the UF reservoir, just below the ceramic membranes, which allowed for membrane scouring. The UF system was equipped with a peristaltic pump to extract the treated permeate out of the system and to backwash the membranes. The pump was provided with a variable frequency drive, so different flow rates could be established for either a permeate production at 24 L h^−1^, or a backwash flow rate at 47 L h^−1^ (i.e., setting operational fluxes of 23 and 45 L m^−2^ h^−1^ for the permeate production and backwash, respectively). The membrane backwash was applied for 60 s every 10 min, while a temperature and pressure gauge were utilised for monitoring the temperature and pressure (ELPRO Lepenik & Co., Slovenia). The permeate obtained from the UF membranes was stored in a permeate tank from which it was subsequently pumped to an RO membrane filtration unit for further treatment. A booster pump was used to drive the UF permeate through a single RO membrane element unit (Lenntech type CSM RE 4021-BE) with a nominal salt rejection capacity of 99.7%. The RO membrane unit had an effective membrane area of 3.3 m^2^ and operated in a crossflow mode at a flow rate of 230 L h^−1^ (i.e., a flux of 70 L m^−2^ h^−1^) and at a pressure of 8 bars. The RO membrane element was inserted into a compact pressure housing vessel and the pressure was monitored in the feed line by installing a pressure gauge (ELPRO Lepenik & Co., Slovenia). An additional pressure gauge was added in the concentrate line to monitor the changes in the transmembrane pressure and the RO permeate recovery was adjusted to 56% by means of a butterfly valve located at the membrane’s feed line. Three sampling points were provided as follows: in the feed, in the concentrate, and in the permeate lines.

Other components placed in the pilot system included (i) electrical control panels for pilot system operation (#7, Fig. 2), (ii) a compressor (#8, Fig. 2), and (iii) an air-conditioning system (#9, Fig. 2).

### Experimental procedure

The SS was obtained from a lined septic tank (3 × 3 × 4 m) located in the proximity of the GJU experimental site in the Jordan Valley area of Jordan. The septic tank collected and partially treated waste that was primarily generated by a nearby hospital, with the SS emptying frequency varying between 2 and 3 months. A groundwater well was located approximately 50 m away from the septic tank and, on three different occasions (three different batches to suit this research), the SS was transported to the experimental site by an SS pump-out truck. The SS was then conveyed to the receiving reservoir (#1 in Fig. 2), before subsequently being pumped to the coagulation and flocculation unit (#2 in Fig. 2). Within this unit, the suspended solids were destabilized and flocculated by adding ferric chloride and powder polymer (“Septage sludge intake station and coagulation and flocculation unit” section). The required dosages of ferric chloride and polymer were determined on site by carrying out jar-test experiments for each individual SS batch. A ferric chloride solution was added to a 1-L beaker containing the SS. The solution was stirred for 5 min at 100 rpm. After destabilizing the solids, the polymer was introduced while gently stirring the solution for 10 min at 50 rpm. The SS flocs were then allowed to settle for 10 min. After settling, the jar’s contents were passed through a 0.5-mm sieve (same diameter as the sieve contained in the MD unit described in the “Mechanical dewatering unit” section). The ferric chloride and polymer optimal doses were determined by evaluating the sieve sludge retention efficiency and weighing the mass of the dewatered SS retained on the filter. In addition, visual inspections were carried out to assess both the consistency of the SS flocs, and, the remaining turbidity of the supernatant. SS flocs with a slimy appearance and consistency indicated a polymer overdose. The polymer optimal dose determination was carried out with the assistance of the polymer manufacturer (Specialized Water Technologies, Amman, Jordan).

The flocculated SS was conveyed to the MD unit (#3, Fig. 2) by gravity. Two products were obtained out of the MD process: (i) the dewatered SS and (ii) the filtrate. The dewatered SS was manually collected in PP vessels and transferred to the MW drying unit (#4, Fig. 2), whilst the liquid stream (filtrate) was further treated in the membrane filtration system (#5 and #6, Fig. 2). The dewatered SS (SS cake) obtained from the MD unit was manually collected and weighted. Then, the dewatered SS was placed in the MW drying unit holding vessels. The dewatered SS was irradiated in the MW units in batches of 0.5 kg each, with the power set at 1.5 kW. The MW drying units were operated long enough (for approximately 26 min) to provide sufficient energy for both inactivating the pathogens and dehydrating the SS. The MW irradiation caused the SS temperature to increase, which led to the subsequent sanitization of the sludge and evaporation of the water. The evaporated water (vapour) was directed to a filtration system for odour control by means of a ventilator. The resulting condensate was collected in the UF reservoir together with the filtrate produced by the MD unit. These liquid streams, both the condensate and the filtrate, were filtered by the UF ceramic unit. The resulting permeate was further treated (polished) by the RO unit.

**Fig. 3:**
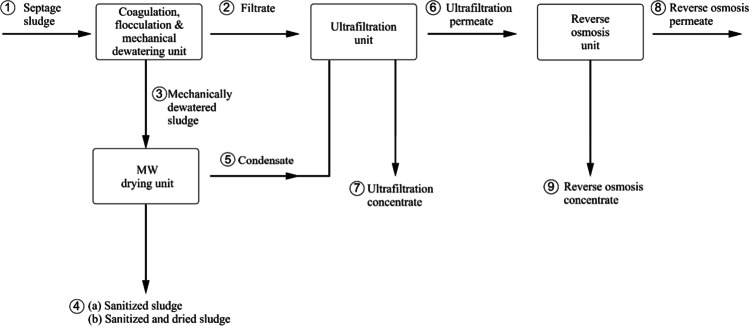
Process flow diagram indicating the sampling points as follows: (1) septage sludge; (2) filtrate; (3) mechanically dewatered sludge; (4.a) sanitized sludge; (4.b) sanitized and dry sludge; (5) condensate; (6) UF permeate; (7) UF concentrate; (8) RO permeate and; (9) RO concentrate

Several different samples were collected to represent the entire treatment process as numbered in Fig. 3: (1) SS; (2) filtrate; (3) mechanically dewatered sludge; (4a) sanitized sludge; (4b) sanitized and dried sludge; (5) condensate; (6) UF permeate; (7) UF concentrate; (8) RO permeate; and (9) RO concentrate. Three independent evaluations (batches) were carried out, with one individual sample taken from each batch at the sampling points indicated in Fig. 3. The analytical determinations conducted on the collected samples are presented in the “Analytical determinations” section that follows.

### Analytical determinations

The samples, as described in Fig. 3, were collected in polyethylene bottles tightly sealed with a cap, then stored in a refrigerator. The reservoirs containing sludge at low DS concentrations (such as the raw SS reservoir) were well mixed before sampling with either air or a recirculation pump, carried out to reduce the possibility of solids settling and, thus, allowing to obtain a representative sample. The reservoirs containing sludge at high DS concentrations (such as the dewatered SS reservoir) were mixed manually before sampling. The sludge samples were taken approximately one hour after each particular individual treatment process was initiated, then were properly preserved to allow for the specific physical–chemical parameters to be determined. The physical–chemical characteristics of the collected samples were analysed to determine the levels of DS, volatile solids (VS), *E. coli*, chemical oxygen demand (COD), soluble COD (sCOD), total nitrogen (TN), total phosphorus (TP), PO_4_^3−^, NH_4_^+^, potassium (K), heavy metals (chromium (Cr), nickel (Ni), cadmium (Cd), zinc (Zn), lead (Pb), and mercury (Hg), together with an elemental analysis to include the elements of carbon (C), hydrogen (H), nitrogen (N), and oxygen (O). The analytical determinations were carried out at an external laboratory in Jordan (NaraTech LABs, Amman, Jordan), with the samples analysed within two days of sample collection. Approximately, nine samples were collected for each batch (i.e., 27 samples in total). Table 1 describes the different analytical determinations carried out on the different samples, while the methods conducted for the analytical determinations are shown in Table 2. The pH and the electrical conductivity (EC) of the samples were analysed immediately after the samples were taken using a portable pH (ProfiLine pH 3110, WTW, Germany) and an EC meter (GMH 3430, Greisinger, Germany). The final DS concentration of the dried sludge was calculated by determining the mass change of the samples (as they were being dried in the MW unit). The mass was measured using a single point-cell (scale) installed within the MW cavities. The higher heating value (HHV), or gross energy, was calculated from the elemental composition of the sludge as per Friedl et al. (2005). All the results were reported in consideration of the average values and standard deviations from the three different batches. Each SS batch required 2 days to be fully processed; thus, each batch corresponded to two days of system operation. The following physical–chemical parameters (*E. coli*, TP, and PO_4_^3−^) were calculated by conducting mass balances (i.e., those parameters were not analysed).

**Table 1:** Monitoring parameters

Sample point and ID		DS	VS	*E.coli*	COD	sCOD	TN	NH_4_^+^	TP	PO_4_^3−^	K^+^	pH	Conductivity	Heavy metals	CHNO
1	Septage sludge	•	•	•	•	•	•	•	•	•	•	•	•		
2	Sludge filtrate	•	•		•	•	•	•	•	•	•	•	•		
3	Mechanically dewatered sludge	•	•	•	•		•	•			•	•		•	•
4.a	Sanitized sludge			•											
4.b	Sanitized and dried sludge	•		•											
5	Condensate				•		•	•	•	•		•			
6	UF permeate	•		•	•		•	•	•	•	•		•		
7	UF concentrate	•			•	•	•	•	•	•	•	•	•		
8	RO permeate	•		•	•		•	•	•	•	•	•	•		
9	RO concentrate	•		•	•		•	•	•	•	•	•	•

**Table 2:** Analytical methods for the determination of the sludge physical–chemical characteristics

Parameter	Method
*E. coli*	ISO9308:2014
COD	SM5220C
sCOD	SM5220C
TN	EL-WL-SOP-003
TP	ST066
PO_4_^3−^	SM4110
NH_4_^+^	SM4110
Metals	ST066
TS	SM-2540D
VS	SM-2540E
CHNO	ST078

## Results and discussion

### Performance of the coagulation/flocculation and mechanical dewatering units on SS treatment

**Fig. 4:**
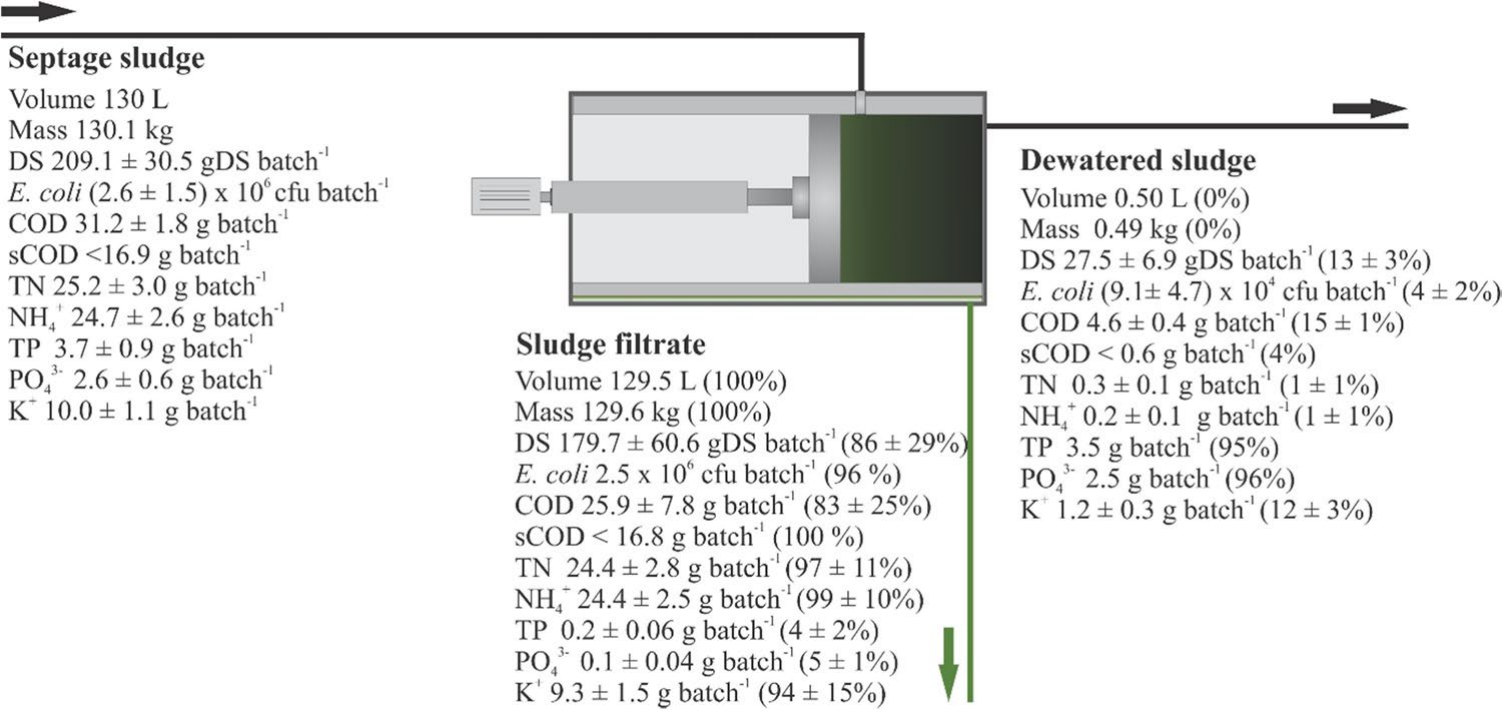
Mass balance of the coagulation/flocculation unit and mechanical dewatering

The characteristics of the SS before being flocculated (influent SS) and after MD are presented in Table 3. The particle destabilization of the sludge was carried out by dosing ferric chloride at 120 mg L^−1^ followed by rapid mixing, while the flocculation of the sludge was achieved by adding the polymer at a concentration of 50 mg L^−1^ followed by slow mixing. The pilot unit was operated in a batch mode; an overall mass balance of the coagulation/flocculation and MD unit is presented in Fig. 4.

**Table 3:** SS physical–chemical characteristics before and after coagulation/flocculation and mechanical dewatering

Parameter	Units	Septage sludge (influent)	Filtrate	Mechanically dewatered sludge
DS	%	0.16 ± 0.02	0.14 ± 0.05	5.62 ± 0.14
VS	%	47.1 ± 9.60	50.7 ± 1.18	31.8 ± 0.67
Density^b^	g cm^−3^	1.00	1.00	1.02
pH	-	7.5 ± 0.21	7.3 ± 0.42	6.5 ± 0.22
EC	µS cm^−1^	(2.94 ± 0.24) × 10^3^	(3.05 ± 0.21) × 10^3^	-
*E. coli*^c^	CFU g^−1^	(2.00 ± 1.15) × 10^1^	1.93 × 10^1a^	(1.87 ± 0.97) × 10^2^
COD^c^	mg g^−1^	0.24 ± 0.01	0.20 ± 0.06	9.48 ± 0.75
sCOD^c^	mg g^−1^	< 0.13	< 0.13	-
TN^c^	mg g^−1^	0.19 ± 0.02	0.19 ± 0.02	0.56 ± 0.27
NH_4_^+c^	mg g^−1^	0.19 ± 0.02	0.19 ± 0.02	0.40 ± 0.26
TP^c^	mg g^−1^	0.03 ± 0.01	0.001 ± 0.0005	7.25^a^
PO_4_^3−c^	mg g^−1^	0.02 ± 0.005	0.001 ± 0.0003	5.10^a^
K^+c^	mg g^−1^	0.08 ± 0.01	0.07 ± 0.01	2.40 ± 0.51
*E. coli*^d^	CFU gDS^−1^	(1.2 ± 0.7) × 10^4^	1.4 × 10^4a^	(3.3 ± 1.7) × 10^3^
COD^d^	mg gDS^−1^	149.2 ± 8.8	144.0 ± 43.2	168.7 ± 13.4
sCOD^d^	mg gDS^−1^	< 80.8	< 93.6	-
TN^d^	mg gDS^−1^	120.6 ± 14.5	135.8 ± 15.5	10.0 ± 4.8
NH_4_^+d^	mg gDS^−1^	118.1 ± 12.4	135.6 ± 14.04	7.2 ± 4.6
TP^d^	mg gDS^−1^	17.8 ± 4.1	0.9 ± 0.3	129.0^a^
PO_4_^3−d^	mg gDS^−1^	12.6 ± 3.1	0.7 ± 0.2	90.9^a^
K^+d^	mg gDS^−1^	47.7 ± 5.2	51.8 ± 8.2	42.6 ± 9.1

^a^Calculated from the mass balance

^b^The density of sludge obtained from Radford et al. (2014)

^c^The concentrations of chemical compounds or colony-forming units were expressed as mg or CFU per g of solution

^d^ The concentrations of chemical compounds or colony-forming units were expressed as mg or CFU per g of DS (dry mass basis)

The DS concentration in the influent SS, as shown in Table 3, was 0.16% on average. This value agrees with the literature on sludge derived from septic tanks in Jordan (Halalsheh 2008). For example, Halalsheh (2008) reported average DS content values of 0.2% in SS samples examined in the winter and 0.5% in those examined in the summer. The low DS values reported in the SS could be partly attributed to a pilot test period (winter season) characterized by higher precipitation rates than during the summer season. Brackish groundwater intrusion into septic tanks may also contribute to such low DS values depending on the rain precipitation patterns, water table levels, and construction characteristics of the septic tanks (Halalsheh 2008). The EC measured in the influent SS (reported in Table 3) exhibited a value of 2.9 × 10^3^ μS cm^−1^. As estimated by applying the software Hydranautics IMS, the presence of NH_4_^+^, PO_4_^3−^ and K^+^ contributed to a conductivity of 1.0 × 10^3^ μS cm^−1^ (approximately 34% of the influent SS conductivity) equivalent to a total dissolved solids (TDS) concentration of 0.03%. Moreover, according to the formula provided by Rusydi (2018) (TDS = *k* × EC considering a correlation coefficient *k* of 0.55), the TDS values of the remaining ions were approximately 0.1%. Thus, these results indicated that the major components of the total DS measured in the SS (0.16%) were dissolved solids (0.13%), meaning that the SS contained a relatively low amount of suspended solids (0.03%). Based on these previous calculations, an eventual brackish groundwater intrusion in the influent SS can be suspected. The intrusion of brackish groundwater in SS tanks in Jordan was confirmed by Halalsheh (2008); the author reported an average TDS concentration of approximately 0.1 and 0.3% in SS samples analysed in the winter and summer period, respectively. Such TDS content in the SS is close to the maximum allowable limit for water reuse for irrigation purposes (TDS < 0.15% or < 1.5 g L^−1^) as stated in the Jordanian standard 893/2006 (Ulimat 2012). Accordingly, if the water content of such SS is intended to be applied in irrigation applications, then it would be safe to apply further measures, such as RO filtration for removing divalent and monovalent ions. The type of wastewater the septic tank is receiving (hospital wastewater and/or laundry wastewater), as well as the strategies followed for emptying the septic tank (removing just the liquid fraction—supernatant—from the septic tanks rather than settled solids) could contribute to the DS content of the SS.

The influent SS was destabilized and flocculated using ferric chloride and polymers, respectively. Upon flocculation, the SS was dewatered using the MD unit. The DS content of the SS went up from 0.16 (influent SS) to 5.6% (dewatered SS). The influent SS was concentrated approximately 35 times, which indicates the fairly good MD unit performance. However, the DS concentrations obtained in the dewatered SS were much lower than expected considering both the initial low DS content of the influent SS, and also the high presence of TDS that ended up in the filtrate. As shown in the mass balance presented in Fig. 4, approximately 13% of the DS initially present in the influent SS were retained by the filter screen of the MD unit, while the rest of the DS content passed through the sludge filtrate. The relatively high content of DS in the sludge filtrate, as previously reported, could be due to the high presence of TDS (not measured in this research but estimated from the EC determinations). In addition, the passage of such a large amount of solids into the filtrate could also be due to the relatively large aperture size of the MD unit sieve (approximately 0.5 mm). Due to the sieve having such a large aperture size, negligible clogging of the sieve was observed.

The coagulation/flocculation process could promote the simultaneous precipitation and removal of nutrients (such as phosphate) and organics sequestered in the solid cake. For instance, as shown in Fig. 4, the total mass of TP and PO_4_^3−^ was much higher in the dewatered sludge (3.5 g of TP – 95% of the influent content, and 2.5 g of PO_4_^3−^ – 96% of the influent content) when compared to the sludge filtrate (0.2 g of TP – 4% of the influent content and 0.1 g of PO_4_^3−^ – 5% of the influent content). On the other hand, as shown in Fig. 4, 99% and 94% of the influent content for the NH_4_^+^ and K^+^, respectively, remained in the liquid fraction. The results presented in Table 3 and Fig. 4 indicate that the soluble constituents (except for PO_4_^3−^) present in the influent SS ended up in the filtrate (liquid fraction), while the particulate constituents were entrapped within the solid matrix as expected.

The MD process allowed for the removal of approximately 99.6% of the initial SS water content. Thus, the subsequent MW drying unit would receive (i) less volume of sludge requiring drying; and (ii) sludge with a much lower water content (i.e., much higher initial DS%) compared to the influent SS. In this study, each batch of sludge to be MW dried had a volume of approximately 0.5 L at a DS% of 5.6% compared to the SS influent batch before dewatering, with an initial total volume of 130 L at a DS of 0.16%. As such, the MW power output needed to meet the treatment objectives (such as the final DS% of the dried sludge) can be optimized. This will have a positive impact on the overall capital costs, operational costs, and footprint needs of the system. These results demonstrate that pre-treating the sludge using the MD process had a positive effect on the subsequent MW drying process. In addition, a liquid stream is produced (filtrate) that can be further polished to obtain different water qualities for further water reclamation applications.

### Performance of the MW drying unit on sludge sanitization, drying, and suitability of the sanitized sludge for land applications.

This section presents and evaluates the effects of MW radiation on the sanitization and drying performance of the dewatered SS retained by the MD unit. The sanitization and drying performance of the MW system was assessed by monitoring the pathogenic content of the sludge and the changes in sludge mass, respectively, as a function of the exposure time at predefined MW power outputs. The final products resulting from the MW treatment, including the treated (sanitized and dried) sludge and the water vapour (condensate) during the MW heating, were characterized (Table 6 and Table 5, respectively) for evaluating the presence/absence of contaminants that may eventually have a negative effect on crops. In addition, other dried sludge components were characterized, such as the calorific value and the elemental composition of selected elements (Table 4).

**Table 4:** Elemental composition and calorific value of the mechanically dewatered sludge on a dry mass basis

Parameters	Units	Mechanically dewatered sludge
C	%	30.7 ± 2.6
H	%	5.2 ± 1.1
N	%	5.1 ± 0.9
O	%	28.3 ± 1.9
HHV	MJ kg^−1^	16.4

#### Sanitization of sludge using MW-based technology

**Fig. 5:**
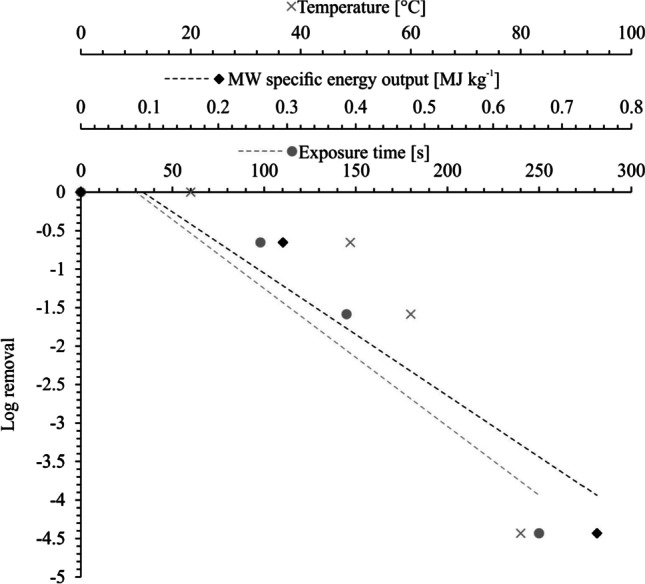
Log removal of *E. coli* as a function of exposure time, MW specific energy output, and temperature

Figure 5 illustrates the MW performance for the sanitization of the MD sludge, which was evaluated by determining the *E. coli* log removal as a function of the exposure time and MW specific energy output (i.e., MW power output per unit of initial sludge mass). In addition, Fig. 5 also indicates the change in the sludge temperature as a function of the exposure time.

##### The effect of temperature on pathogen reduction

As shown in Fig. 5, the *E. coli* log reduction increased linearly as a function of the exposure time, and temperature. An exposure time of 250 s was required to achieve a 4.4 *E. coli* log removal. In other words, 250 s were required to reduce the *E. coli* count from 1.87 × 10^2^ cfu g^−1^ (or 3.3 × 10^3^ cfu gDS^−1^) to below the detection limit (0.98 × 10^2^ cfu g^−1^ or 0.17 × 10^3^ cfu gDS^−1^). This corresponded to a final temperature of 80 °C. The removal of pathogens (expressed as *E. coli* in this study) was obtained within a short exposure time, producing an observation that agrees with the literature. For instance, Mawioo et al. (2016a) evaluated the *E. coli r*eduction in fresh FS, derived from pit latrines. The authors observed a reduction of the *E. coli* count below the detection limit after only one minute of exposure at MW power outputs of 1.08 kW and 1.55 kW. Several other studies have been conducted on a wide range of sludge, including blackwater sludge, waste activated sludge, centrifuged waste activated sludge, SS and fresh FS, reporting similar observations (Hong et al. 2004, 2006; Mawioo et al. 2016a, b; Pino‐Jelcic et al. 2006). The removal of pathogens could be attributed to the molecular excitation caused by MW irradiation of the sludge. This led to an increase in sludge temperature, which is a critical factor influencing microbial destruction in MW applications. Complete microbial destruction occurs at temperatures above 70 °C (Hong et al. 2004). Thus, the complete inactivation of *E. coli* achieved at a temperature of 80 °C (as shown in Fig. 5) agrees with previously reported microbial removal temperatures (Hong et al. 2004). A temperature of 70 °C has also been reported as sufficient for inactivating other pathogens such as *Ascaris eggs* (Mawioo et al. 2016a). In addition, the excitation of the molecules caused by the MW irradiation of the sludge could have damaged the cells through non-thermal effects (Banik et al. 2003; Hong et al. 2004). The non-thermal effects, unlike the thermal ones, cannot be directly measured. Such effects are suspected to result from the interaction between the polarized macromolecules and the oscillating electric field generated within the MW cavity. This interaction has been reported to wriggle the polarized molecules, causing cell breakages that lead to cell death and the consequent release of intracellular material (Herrero et al. 2008). Ultimately, in both thermal and non-thermal microbial destruction cases, it has been observed that the degree of cell damage increases when the MW specific energy output increases.

##### The effect of MW specific energy output on pathogen reduction

As shown in Fig. 5, an MW-specific energy output of 0.75 MJ kg^−1^ (0.2 kWh kg^−1^) was required to achieve complete pathogen destruction. The energy consumption required to reduce the *E. coli* count below the detection limit is in good agreement with the literature. For example, Karlsson et al. (2019) reported energy consumptions ranging from 0.33 to 3.1 MJ kg^−1^ (0.09 to 0.9 kWh kg^−1^) for complete *E. coli* inactivation. The differences in the specific energy outputs required to achieve complete pathogen destruction may be attributed to several factors, including the selected pathogen indicators, the physical–chemical properties of the sludge (e.g., mass, density, specific heat, ionic content, dielectric properties), operational parameters, and specific MW-related settings—such as the MW operating frequency, MW power outputs, and overall MW system design features, among others. For example, Hong et al. (2016), when microwaving coal, reported that the MW power output was one of the most critical factors to consider when designing an MW drying system. The power output had a strong influence on how fast the material was heated up. Thus, the temperature required for sludge sanitization can eventually be reached more quickly by applying a higher MW power output. Additionally, this would lead to lower specific energy outputs for achieving the desired goal. The applied MW power output for achieving complete sludge sanitization would also depend on the sludge’s initial DS content. In other words, the lower the net amount of water present in the sludge, the lower the specific heat capacity, meaning that less energy would be required to reach the sanitization temperature with the corresponding pathogen destruction (Mawioo et al. 2017). The pathogen destruction may also vary according to the specific indicators used to evaluate the MW system’s sanitization performance. For example, Mawioo et al. (2017) reported that the MW-specific energy output required to inactivate *E. coli* in SS was much larger compared to the energy required to inactivate both *Enterococcus faecalis* and *Staphylococcus aureus*. Furthermore, the type of sludge being irradiated also exhibited different pathogen-removal efficiencies (Mawioo et al. 2017). In addition, the specific energy output required to sanitize the material would also depend on the MW system design (Vadivambal and Jayas 2010) whereas the most important design issue associated with pathogen destruction is the uniformity of the electromagnetic energy distribution within the material. An uneven temperature distribution would lead to incomplete pathogen destruction, which raises issues for the safe handling and potential reuse of the dried sludge (Vadivambal and Jayas 2010). The occurrence of hot and cold spots can be reduced by varying the MW frequency and/or by the provision of a turntable and/or an agitator in the MW irradiation cavity (Jones et al. 2002), carried out in this study.

The results presented here confirm that MW irradiation can be applied for sludge sanitization, with the MW system capable of achieving a complete reduction of *E. coli* at the evaluated conditions and at a relatively low MW-specific energy output (and short exposure times).

#### Drying of sludge using MW-based technology

**Fig. 6:**
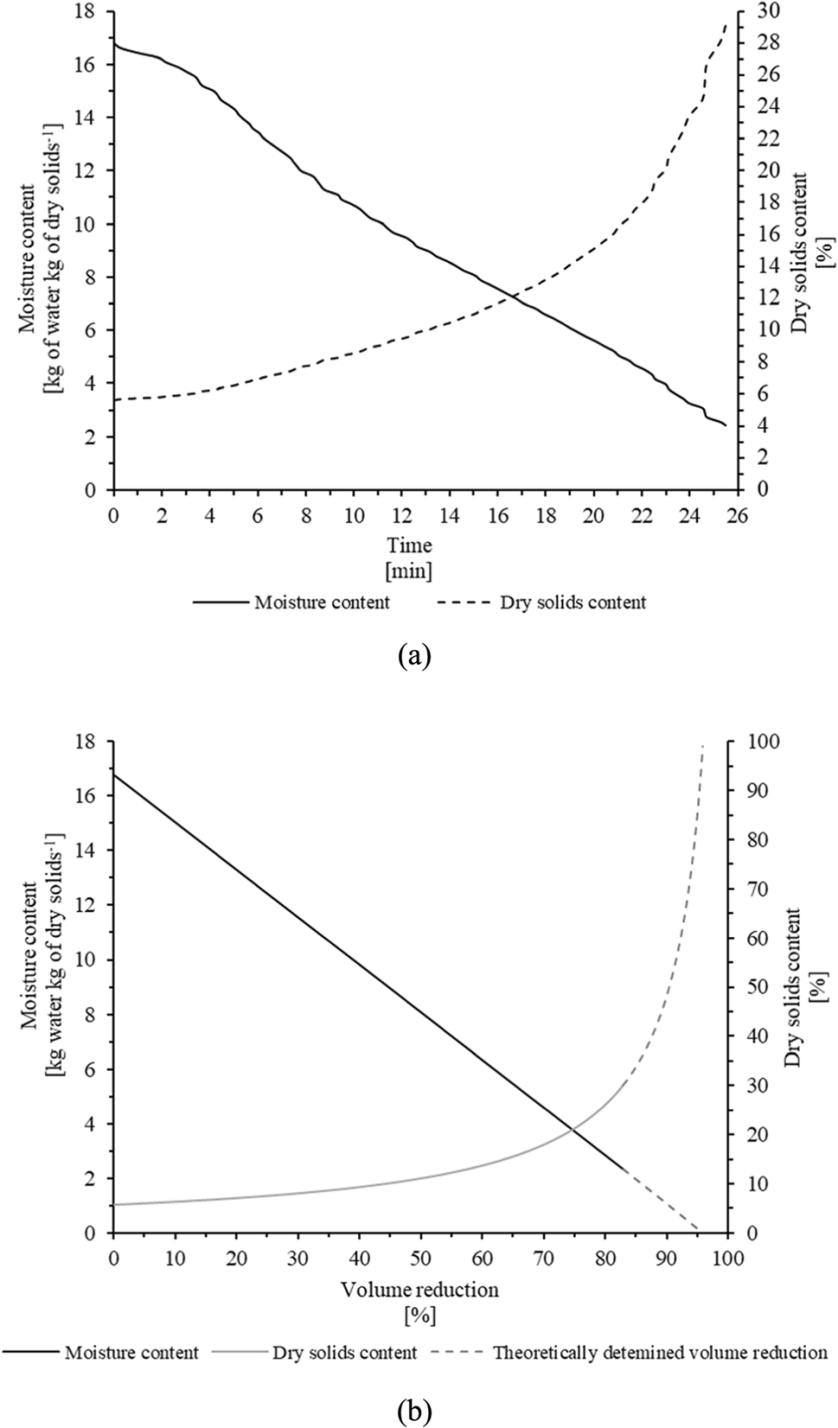
Sludge moisture content and dry solids content as a function of **a** exposure time and **b** sludge volume reduction

**Fig. 7:**
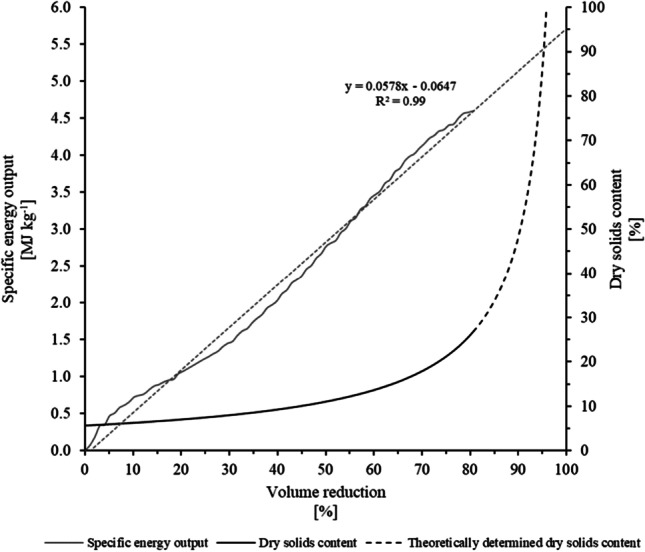
Specific energy output and sludge DS content as a function of the sludge volume reduction

Figure 6a illustrates the changes in sludge moisture content (dry solids content) as a function of the MW irradiation exposure time at an MW power output of 1.5 kW. The MW irradiation chamber was loaded with 0.5 kg of sludge at a DS concentration of 5.6%, so an initial power/mass ratio of 3 kW/kg was applied. This value corresponds to the maximum initial power/mass ratio applied in previous MW pilot-scale evaluations (Kocbek et al. 2020; Mawioo et al. 2017). The sludge was irradiated for approximately 26 min, and the DS content increased from 5.6 to 30.0% (i.e., sludge moisture content decreased from 16.8 to 2.4 kg of water kg of dry solids^−1^). As a result of such sludge dehydration, the sludge volume decreased by approximately 83%, as shown in Fig. 6b. This corresponds to an energy consumption of 4.6 MJ kg^−1^ (1.3 kWh kg^−1^). Notably, a higher degree of volume reduction could still be achieved by simply prolonging the irradiation exposure time. However, a substantial increase in energy expenditure would occur. Such extrapolation is indicated in Fig. 7 (provided that the system’s energy expenditure is proportional to the exposure time). So, an MW-specific energy output (defined in Sect. 3.2.1) of 5.5 MJ kg^−1^ (1.5 kWh kg^−1^) would be required to achieve a volume reduction of approximately 95% that also corresponds to a DS content of higher than 95%. Such a specific energy output calculation does not consider the efficiency for converting electrical energy into electromagnetic energy (i.e., the MW generator energy efficiency). The MW generator efficiencies that utilize magnetrons range between 50 and 72% and between 80 and 90% at MW frequencies of 2,450 MHz and 915 MHz, respectively (Evans and Hamlyn 1996). As such, the energy expenditures (cost) for drying the sludge would directly depend on the type of energy (MW frequencies) used by the system. According to Kocbek et al. (2020), MW systems operated at a power output of 1.5 kW have an MW generation efficiency of 57%. Considering this value, the specific energy consumed during the drying process would be almost double that reported in this study (8.6 MJ kg^−1^ or 2.4 kWh kg^−1^). Moreover, as indicated in the “Sanitization of sludge using MW-based technology” section and also as reported by Kocbek et al. (2020), the higher the MW power output, the higher the power absorbed by the material being dried, minimizing the possibility of energy losses occurring somewhere in the system and, therefore, increasing the energy efficiency of the MW drying system. Thus, operating the system at the highest nominal power capacity maximizes the MW energy efficiency and leads to a reduction in the specific energy output required to dry the material. An increase in the rate at which the energy is delivered has a strong influence on the MW generation efficiency. In addition, the higher the energy absorbed by the material per unit of volume, the higher the water evaporation rate, meaning shorter required irradiation exposure times (Bennamoun et al. 2016; Chen et al. 2014). In other words, the energy expenditure of MW technology is partially dependent on the MW operational conditions, so it may be optimized by varying the sludge mass or the MW power output settings.

Furthermore, the energy contained in the vapours/condensate (produced when evaporating the sludge’s water content) may eventually be used to increase the temperature of the dewatered sludge before it is introduced into the MW irradiation cavity. Thus, by reducing the overall energy demand for drying the sludge, the specific energy output can be reduced by approximately 10% (Kocbek et al. 2020). In addition, energy can also be recovered/obtained from burning the MW-dried sludge via a combustion process. The elemental compositions of the dried sludge for C, H, N, and O were determined and presented in Table 4. The gross calorific value of the dried sludge was determined from the elemental composition determination and is also presented in Table 4. If each batch is MW-dehydrated from an initial sludge mass of 500 g (at a 5.6% DS) to a final dried sludge mass of 30 g (at 90% DS), then an additional energy recovery of 0.5 MJ (i.e., 30 g at 16 MJ kg^−1^) or specific energy output savings of 1 MJ kg^−1^ can be achieved by burning the resulting dried sludge. Therefore, considering the recovery of energy from both the vapour/condensate obtained during the MW drying process, as well as from the combustion of dry sludge, the specific energy output may be decreased from approximately 5.5 to 4 MJ kg^−1^ (i.e., from 1.5 to 1.1 kWh kg^−1^). In addition, the MW drying energy expenditures may be further reduced by employing more effective MD systems, resulting in a sludge that can be MW irradiated with a higher initial DS content. As a result, the specific energy output may be considerably reduced, while also increasing the energy recovery possible from dry sludge combustion. Nevertheless, the elemental characterization of the dried sludge indicated C and N fractions of 30.7 and 5.1%, respectively. This may be associated with the formation of nitrogen oxides (NO_X_) and carbon dioxide (CO_2_), which may have a negative impact on the environment. It may be required, therefore, though depending on local regulations, to implement additional filters for the treatment of the exhaust gasses formed during combustion. As such, this may result in an increase in the capital and operational costs required for running the system. Recovering energy from the generated vapours/condensate alone, however, may not exhibit such inconveniences. In addition, the implementation of renewable energy sources for powering the MW drying system, such as photovoltaic cells, may also be a possibility (as discussed in more detail in the “Outlook on the use of integrated technologies in the treatment of septage sludge” section).

The results presented in this section indicate that, at the evaluated conditions, the DS content of the sludge was increased up to approximately 30%, corresponding to a volume reduction of 83%. However, by simply increasing the irradiation exposure time, a DS content of 95% can be achieved, as also reported by other authors (Chen et al. 2014; Kocbek et al. 2020; Mawioo et al. 2016b). MW-specific energy expenditures of 5.5 MJ kg^−1^ (1.5 kWh kg^−1^) are required for drying the sludge (up to 95% DS), which is comparable to energy expenditures on conventional sludge dryers (Kocbek et al. 2020). In addition, a reduction of MW-specific energy output may be further achieved by employing more efficient MD systems, by using more efficient MW systems, and by optimizing operational parameters that include the MW power/mass ratio, among others (Chen et al. 2014; Mawioo et al. 2016b). In addition, the energy expenditures can be further reduced by recovering the energy from the condensate/vapour and from burning the dried sludge.

The MW irradiation of the sludge produced a vapour stream containing mostly water and some volatile compounds originally present (Deng et al. 2009). Such gas stream condensates before reaching the odour control filters, then the condensate is directed to the UF reception tank. Following this, the condensate was characterized to evaluate the resource-recovery possibilities, with the results presented in Table 5. Results show that the condensate derived from the sludge drying process is pathogen-free. Overall, the values reported in this study are comparable with the condensate characteristics reported in previous studies focused on MW drying of SS (Mawioo et al. 2017). However, the average concentration of TN reported in this study—33.4 mg L^−1^—is lower than reported by Mawioo et al. (2017), which was recorded as 213 mg L^−1^. This could be attributed to an increase in the pH during this study, which could affect the speciation of ammonia from NH_4_^+^ to NH_3_. Ammonium in its gaseous phase (NH_3_) could have become volatilized during condensation and/or the sampling period. It should be considered, therefore, that the composition of the condensate varies considerably depending on the origin of the sludge and on the technology employed for drying (Deng et al. 2009; Karwowska et al. 2016). For example, Karwowska et al. (2016) reported COD, NH_4_^+^, and PO_4_^3−^ concentrations in the condensate of conventional thermal drying systems ranging from 109 to 2,240, from 92 to 343, and from 0.5 to 7 mg L^−1^, respectively.

**Table 5:** Condensate physical–chemical characteristics

Parameters	Units	Condensate
COD	mg L^−1^	565.0 ± 7.8
TN	mg L^−1^	33.4 ± 8.2
NH_4_^+^	mg L^−1^	32.7 ± 7.6
TP	mg L^−1^	1.8 ± 0.1
PO_4_^3−^	mg L^−1^	< 0.6
pH	-	8.8 ± 0.02

#### Suitability of the MW processed sludge for land application

Table 6 describes the requirements/regulations set out by the United States Environmental Protection Agency (USEPA) and by the European Union (EU) for the reuse of treated sludge for land applications, together with the results obtained in this study. In doing so, Table 6 indicates that the sanitized sludge treated using the MW system fulfils the current standards regarding *E. coli* concentration in the USEPA guidelines. Additionally, the sludge’s vector attraction requirements must also comply with the USEPA, as must the presence of heavy metals in the treated sludge. With respect to the vector attraction, a 38% reduction of the initial VS amount is required to fulfil such requirements (USEPA 1994). In the EU, standardized regulations for vector attraction requirements have not yet been established. However, according to the European Environment Agency, a volatile solids/dry solids (VS/DS) ratio below 0.6 is recommended (Bresters et al. 1997). As shown in Table 6, a VS/DS ratio of 0.3 was reported for the dehydrated sludge. However, the MW system was not able to reduce the original VS content, as was also reported by Mawioo et al. (2016a). The total amount of VS remained unchanged before and after the MW drying; however, if a sufficient reduction in the net amount of water is achieved (meaning the potential for microbial growth is reduced), then the sludge may be safely applied to land applications with respect to crop sensitivity issues. According to the USEPA, this can be achieved when drying the material by up to a DS content of at least 90%. Such levels of DS concentration can be achieved by the MW drying system, although in this study the drying was stopped at a DS concentration of approximately 30% after 26 min of MW irradiation. The potential land application of the sludge may also be limited by the presence of heavy metals such as Hg, Ni, Zn, Pb, Cr, Cd, and Cu. The concentrations of such compounds in this study were considerably below the USEPA and EU standards, as indicated in Table 6. These results ascertain that the sludge can be safely reused in agricultural applications, while also showing that the nutrients that can also be recovered would exhibit additional advantages for enhancing crop productivity.

**Table 6:** Physical–chemical properties for the sanitized sludge compared to the USEPA and EU standards for treated sludge land applications

Parameter		Sanitized sludge	USEPA	EU
**Pathogen requirement**				
*E. coli*	cfu gDS^−1^	n.d.^a^	< 1000	-
**Vector attraction requirement**				
VS/DS	-	0.3 ± 0.1	-	0.6^b^
VS reduction	%	-	38	-
**Metal concentration limitation**				
Cr	mg kg^−1^	117.5 ± 36.8	-	-
Zn	mg kg^−1^	722.2 ± 252.9	2800	4000
Cu	mg kg^−1^	60.0 ± 31.4	1500	1750
Ni	mg kg^−1^	15.2 ± 0.2	420	400
Pb	mg kg^−1^	10.5 ± 0.3	300	1200
Hg	mg kg^−1^	0.2 ± 0.1	17	25
Cd	mg kg^−1^	< 0.0001	39	40

^a^Not detected/below the detection limit

^b^Vector attraction reduction recommended value by the European Environmental Agency

### Performance of the membrane separation system for the treatment of the sludge filtrate and condensate

**Fig. 8:**
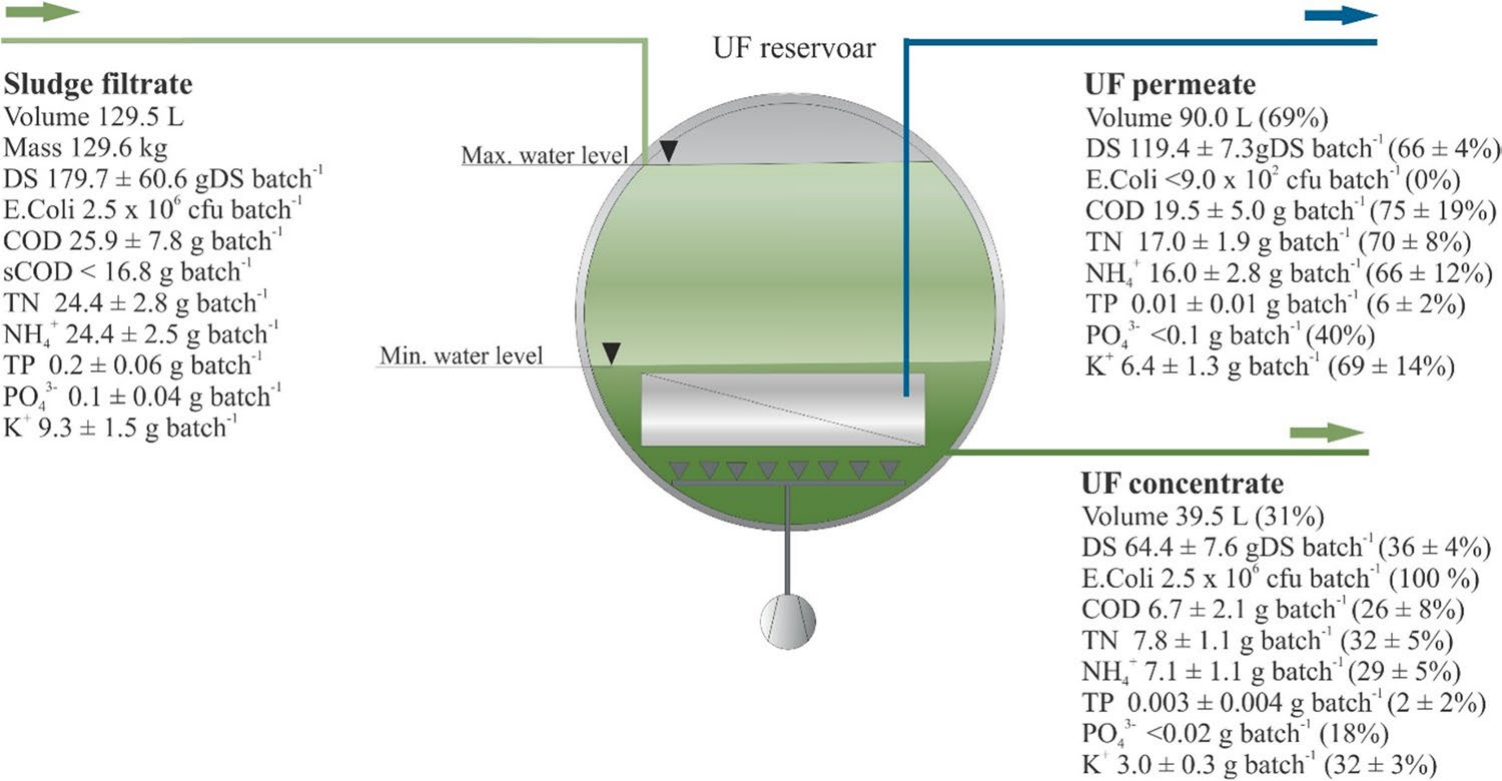
Mass balance of the UF filtration system

The filtrate from the MD unit and the condensate from the MW drying unit were collected in the UF tank and filtered through the UF ceramic membrane system. As indicated in the Fig. 8 schematic, the volume in the UF tank fluctuated (by the use of a level sensor) between a maximum volume of 130 L (the volume of a batch of SS) and a minimum volume of 40 L (minimum volume required to have the ceramic membrane submerged). Table 7 shows the characterization of the filtrate from the MD unit, the condensate from the MW drying unit, and also the UF permeate and concentrate from the UF filtration system. The condensate only contributed to a minor fraction of the entire flow (0.4 L out of 130 L); thus, the contribution of the condensate could be neglected.

**Table 7:** Physical–chemical characteristic of the SS filtrate, condensate, UF permeate and UF concentrate

Parameters	Units	Filtrate	Condensate	UF permeate	UF concentrate
DS	%	0.14 ± 0.05	-	0.13 ± 0.01	0.16 ± 0.02
*E. coli*	CFU mL^−1^	1.9 × 10^1a^	-	< 1.0 × 10^–2^	6.4 × 10^1a^
COD	mg L^−1^	200.0 ± 60.0	565.0 ± 7.8	216.7 ± 55.1	170.0 ± 52.9
sCOD	mg L^−1^	< 130.0	-	-	-
TN	mg L^−1^	188.7 ± 21.6	33.4 ± 8.2	188.7 ± 21.4	196.7 ± 28.9
NH_4_^+^	mg L^−1^	188.3 ± 19.5	32.7 ± 7.6	178.2 ± 31.4	180.3 ± 27.5
TP	mg L^−1^	1.3 ± 0.5	1.8 ± 0.1	0.1 ± 0.03	0.1 ± 0.1
PO_4_^3−^	mg L^−1^	1.0 ± 0.3	< 0.6	< 0.6	< 0.6
K^+^	mg L^−1^	72.0 ± 11.3	-	71.3 ± 14.1	75.7 ± 7.8
pH	-	7.3 ± 0.4	8.8 ± 0.02	7.1 ± 0.2	7.2 ± 0.1
EC	µS cm^−1^	(3.1 ± 0.2) × 10^3^	-	(2.9 ± 0.3) × 10^3^	(3.0 ± 0.1) × 10^3^

^a^ calculated from the mass balance

The UF ceramic membranes had a pore size of 0.08 µm, meaning that only the removal of particles larger than the pore size of the membrane was expected. This included bacteria such as *E. coli*, which were not detected in the UF permeate (concentration below the detection limit of 10 cfu mL^1^). However, marginal differences were noted in the DS content of the filtrate compared to the DS content in the UF permeate and concentrate, confirming that most of the DS content was in the dissolved form (as discussed in the “Performance of the coagulation/flocculation and mechanical dewatering units on SS treatment” section). Furthermore, as shown in Table 7, most of the soluble compounds, such as COD, NH_4_^+^, and K^+^, passed through UF filtration. The mass balance carried out in Fig. 8 also confirmed this observation, with the mass of the soluble compounds possibly being directly related to the volume of either the UF permeate or the UF concentrate produced during UF filtration (i.e., 70% of COD, NH_4_^+^, and K^+^ mass for a particular batch remained in the permeate, while the 30% left remained in the UF concentrate following the permeate/concentrate volume ratio). Moreover, Table 7 and Fig. 8 indicate that both the total phosphorus and orthophosphate were removed from the sludge filtrate but were neither present in the permeate nor in the concentrate. Ferric chloride was added to destabilize the particles in the MD process; therefore, some phosphate precipitation could have occurred in the UF concentrate. When opening the UF tank for maintenance interventions, brown/red precipitates in the pipes, on the surface of the tank, and the surface of the UF ceramic membrane was observed. Thus, some of the TP could remain in such precipitates and not be detected when sampling.

The results of this study indicate that UF filtration has been able to achieve a high removal efficiency for suspended solids and *E. coli*, while allowing for the passage of dissolved components, such as nutrients, and also other salts and minerals present in the filtrate. Consequently, this introduces a severe limitation on the reuse of the UF permeate in irrigation, as the permeate could exceed the maximum allowed concentration for TDS and NH_4_^+^—as stated by the Jordanian standard 893/2006 (Ulimat 2012). Further permeate treatment would, therefore, be desirable if the water is intended for use with irrigation applications. Eventually, UF filtration may be proposed as a pre-treatment process for RO systems, as will now be discussed.

**Fig. 9:**
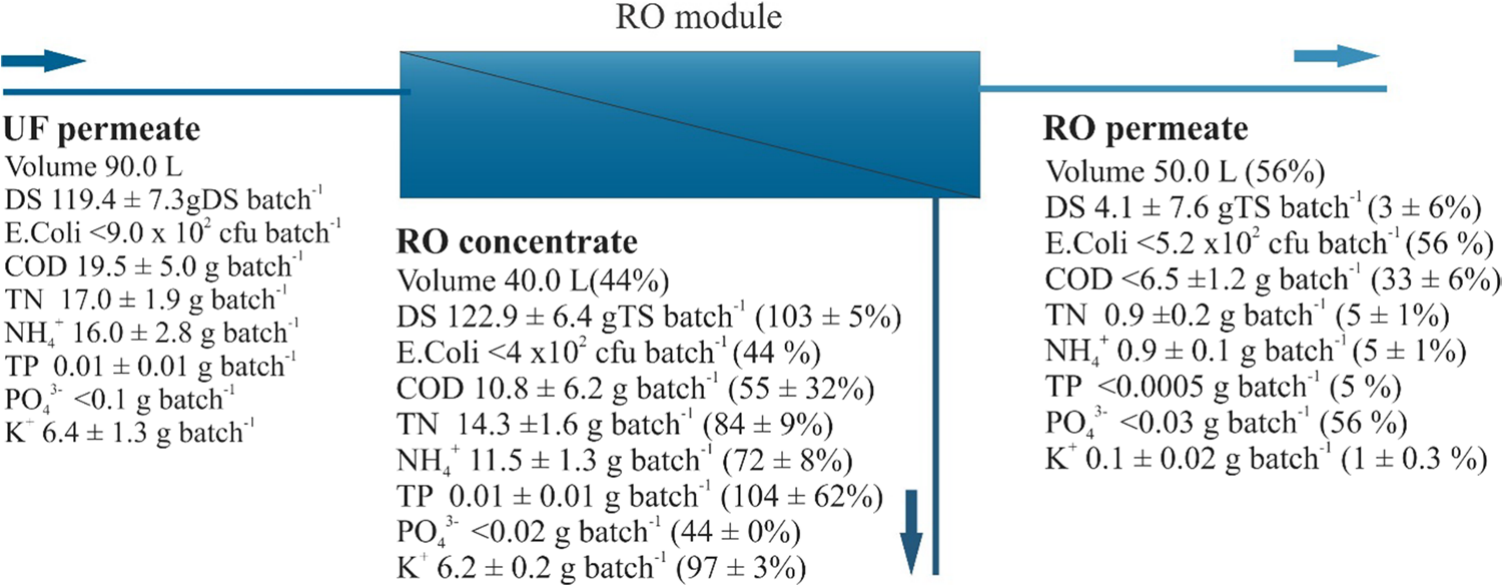
Mass balance of the reverse osmosis system

The UF permeate was collected in an RO reservoir, from which it was directed to an RO filtration unit. The physical–chemical characteristics of the UF permeate (influent to the RO system), RO permeate, and RO concentrate are presented in Table 8. The mass balance of the RO filtration process for an SS batch is presented in Fig. 9. The RO filtration process produces both an RO permeate containing a low concentration of ions and also an RO concentrate consisting primarily of the rejected salts and minerals. This is confirmed in both Table 8 and Fig. 9. As an example of the removal capacities of ions exhibited by the RO filtration system (as illustrated in Fig. 9), the amount of K^+^ in the RO permeate (0.1 g) was much lower than in both the UF permeate (influent to the RO system of 6.2 g) and the RO concentrate (6.4 g), indicating average removal efficiencies of 98%. Table 8 and Fig. 9 also indicate good removal efficiencies for the rest of the valuated compounds. The absence of *E. coli* was also confirmed, both in the RO permeate and concentrate. Furthermore, the RO permeate exhibited a low EC of approximately 121 µS cm^−1^, producing suitable water for reuse in irrigation. In addition, the RO permeate can also be mixed with groundwater to enhance the availability of water resources for irrigation. The TDS removed from the UF permeate were concentrated in the RO concentrate by a factor of approximately two. For instance, the concentration of K^+^ increased from 71.3 in the RO feed to 156.0 mg L^−1^ in the RO concentrate. The RO concentrate exhibited high nutrient concentrations, which can subsequently be recovered by applying several treatment alternatives, including chemical precipitation methods and/or, for example, air-stripping processes that are particularly effective for recovering NH_4_^+^. Furthermore, the energy expenditures of the UF and RO units for treating one batch of SS were 0.55 kWh and 0.06 kWh, respectively. During the research period, membrane fouling was not observed. However, RO fouling is anticipated to occur over an extended treatment period, which will lead to an increase in both operational and maintenance costs.

**Table 8:** Physical–chemical characteristic of the UF and RO permeate and RO concentrate

Parameters	Units	UF permeate	RO permeate	RO concentrate
DS	%	0.13 ± 0.01	< 0.06	0.31 ± 0.002
*E. coli*	CFU mL^−1^	< 1 × 10^–2^	< 1 × 10^–2^	< 1 × 10^–2^
COD	mg L^−1^	216.7 ± 55.1	< 130.0	270 ± 155.6
TN	mg L^−1^	188.7 ± 21.4	17.1 ± 3.4	357.7 ± 39.6
NH_4_^+^	mg L^−1^	178.2 ± 31.4	17.0 ± 2.6	287.5 ± 31.8
TP	mg L^−1^	0.1 ± 0.03	< 0.009	0.2 ± 0.1
PO_4_^3−^	mg L^−1^	< 0.6	< 0.6	< 0.6
K^+^	mg L^−1^	71.3 ± 14.1	1.5 ± 0.4	156.0 ± 5.7
pH	-	7.1 ± 0.2	-	8.6 ± 0.1
EC	µS cm^−1^	(2.9 ± 0.3) × 10^3^	(0.12 ± 0.029) × 10^3^	(5.5 ± 0.5) × 10^3^

The application of UF filtration followed by RO filtration resulted in an efficient method for removing both suspended and dissolved constituents that were originally contained in the SS filtrate after being subjected to an MD process. In assessing the overall treatment performance of the filtration system, treatment efficiencies of over 95% were reported for *E. coli*, DS, TN, TP, NH_4_^+^, and K^+^, as well as approximately 64% for COD and PO_4_^3−^. By following such an approach, the filtrate from the SS after MD could be converted into clean water that is suitable for several reclamation applications, such as crop irrigation, and process water, among others. Additionally, the RO concentrate can be further treated to recover the nutrients contained in the concentrate at relatively high concentrations.

### Outlook on the use of integrated technologies in the treatment of septage sludge

The MD process, aided by the use of particle destabilization and flocculation agents, proved quite successful in increasing the DS content of the SS from approximately 0.16 to 5.6%. However, if the chemical doses are not properly determined, the use of such chemical compounds may result in a reduced dewatering efficiency. In cases where the polymer dose is exceeded, the filtrate may produce a slimy consistency, which may exert a negative impact on subsequent treatment train processes (i.e., the UF system). Both underdosing and overdosing would affect the performance of the MD process, reducing its cost-effectiveness. For SS with DS content below 1%, it is even recommended to bypass the MD process and feed the SS directly into the UF system. At such low DS concentrations, it is envisioned that the coagulation/flocculation process followed by the MD process would be detrimental to the overall process. When the DS concentration of the SS is larger than 1%, the use of an MD process aided by particle destabilization and flocculation is highly justified. However, further research is needed to assess different types of sludge at different initial DS concentrations, which will allow for determining DS concentration cut-off. Furthermore, the MD process had a positive impact on reducing the subsequent MW drying needs (in terms of equipment needs and energy expenditures), required for sanitising and drying the SS.

In addition, the performance of the MW drying (energy expenditure) can be enhanced by employing more efficient MW generators, optimising the MW power output/sludge mass ratio, and recuperating the energy from both the condensate, and also from combusting the dried sludge. However, the exhaust gasses formed during the dried sludge combustion must be monitored and treated if they represent a source of air pollution due to the formation of NOx and CO_2_ gasses.

Furthermore, the UF and RO membrane filtration produced water that could be reused. The SS treated in this study exhibited a high concentration of TDS. Such compounds could have been present in the SS due to the brackish groundwater intrusion in the septic tanks that are commonly observed in the Jordan area (Halalsheh 2008). The reuse of water for irrigation, therefore, would definitely require combining UF and RO filtration in a series. In the absence of brackish groundwater intrusion, combining UF and RO filtration in a series may also be justified when treating SS with a high concentration of ions such as NH_4_^+^ and PO_4_^3−^—dependent on the local water reuse legislation (Forbis-Stokes et al. 2021). In addition, from the RO concentrate, resources such as nutrients may be recovered by either precipitation or stripping. The results of this study indicate that the evaluated combination of technologies was effective for SS sanitisation and drying, while simultaneously recovering resources such as water for irrigation applications.

The energy demands of the evaluated system were also estimated in consideration of the power demand and operational time of each individual component when treating one batch of SS (defined as 130 L of SS). The total energy demand per batch was estimated at 1.8 kWh, which means that the energy demand for a hypothetical sludge treatment system with a treatment capacity of 10 m^3^ of SS per day (i.e., the treatment capacity of the SS collected by a standard 10 m^3^ sludge truck per day) can be extrapolated at approximately 134.1 kWh-day. The MD unit (including the coagulation and flocculation process) would consume 8% of the total energy consumption, while the MW drying unit (including the water-cooling system) would consume approximately 56%. The membrane filtration system (UF and RO) would consume 35% of the total energy. The sludge treatment plant should be designed for being deployed in the operational site with minimum interventions. The system, therefore, should include the possibility for a direct grid connection, in case such a possibility is offered at the operational/deployment site. However, off-grid possibilities should also be provided, such as the option for powering the system with a diesel/petrol generator or an off-grid photovoltaic (PV) system. Considering the high solar irradiance in Jordan (where the system was evaluated), a PV system is applicable for supplying the energy requirements (i.e., a renewable source of energy). In PV systems, the specific energy yield—or simply the yield—describes the energy output of the PV plant over one full year and normalized to the peak power ratings of the plant (kWh kWp^−1^). The specific energy yield, or the energy produced for every kW peak (kWp) of a PV module over the course of a typical year in Jordan, is approximately 1,600 kWh kWp^−1^ (Al-Addous et al. 2017). The treatment of one sludge truck per day (10 m^3^ day^−1^) would require an energy expenditure of approximately 134.1 kWh-day (approximately 50,000 kWh-year). A PV system delivers 0.10 kWp per square meter. Approximately 31.25 kWp would be needed to power such a system, translated into a PV system of approximately 312.5 m^2^. Given that the area needed to accommodate the PV modules is widely available in Jordan, it would be technically feasible to meet the system energy demand by means of renewable energy sources. Two options are envisioned: (i) a non-mobile sludge treatment system can be constructed in a particular location for a specific localized application, which also requires the PV system to be built on location; or (ii) a mobile MSD sludge treatment unit can be projected and the mobile system can be operated with batteries away from the PV energy generation site. The PV modules can generate direct current (DC) and, due to the intermittency of solar supply, they can be combined with an electricity storage system (i.e., batteries). In this sense, the entire energy demand needed to operate the sludge treatment system is transferred via the batteries. The charged batteries can then be placed in the MSD sludge treatment system, which can be deployed in the field. The batteries, once consumed, can be replaced with fully charged batteries and, in such a manner, secure the treatment system operation. In this way, the MSD system can potentially be operated in a sustainable manner, thus, reducing the overall system operation’s carbon footprint. This is especially relevant for applications in Jordan as there is plenty of solar radiation all year round, while Jordan can also not rely on its own supply of fossil fuels for electricity generation, currently importing approximately 96% of its local energy needs (Azzuni et al. 2020). The treatment capacity of the evaluated pilot system can easily be increased to fit such a treatment capacity of 10 m^3^ of SS per day. If larger treatment capacities are needed, several containers can be provided in parallel. However, for large treatment demands in the context of centralized sanitation systems, a larger unit would need to be constructed on the site and without conserving the movable features.

This work indicates that the proposed combination of technologies does enable sludge sterilization and drying, while also providing resource recovery. In addition, the outcomes of this study reveal that MSD-based applications, with designs that enable a quick distribution and installation, may contribute to the provision of sanitation in decentralized and non-sewer contexts (as in emergency situations) that show a rapid generation of sludge. Nevertheless, further improvements are needed, which include (i) optimizing the overall energy consumption of the MSD system; (ii) improving the efficacy of the MD process; (iii) reducing the fouling potential of the membrane filtration system; and (iv) enhancing the MW unit performance by employing an MW system with higher generation efficiency and recovering waste energy for preheating the sludge. Other factors to consider when evaluating the cost-effectiveness of the treatment include the marketability of the sludge-based products and clean water (i.e., the valorisation of such by-products could potentially offset the SS treatment costs). In addition, cost savings from treating and managing the sludge in situ can be achieved (e.g., reduced transportation costs from the generation point to the treatment site and/or its final disposal/reuse location). Moreover, the concept should be evaluated by treating various types of sludge, including fresh FS from pit latrines and other onsite sanitation facilities from diverse locations.

Furthermore, as pointed out by Mawioo et al. (2017), for particular applications—such as in the early stages of sanitation provision after the occurrence of an emergency situation—there are very few sludge treatment options available for treating large amounts of fresh sludge generated over such a short period of time. In situations of this nature, it is important to give priority to the inactivation of pathogens for reducing the sludge moisture content to prevent the propagation of excreta-derived endemics. The sanitization approach requires much lower exposure times when compared to sludge drying (i.e., a much lower energy demand). These sorts of scenarios are ideal for the application of the technology evaluated in this study.

## Conclusion

Three different individual technologies (MD process, MW drying system, and membrane separation processes) were successfully integrated for the treatment of SS in a real application in the Jordan Valley, Jordan. The treatment of SS involved its sanitization and dehydration, while simultaneously producing (recovering) resources such as energy, water, and nutrients. The MD processes were effective in removing approximately 99.6% of the initial SS water content, concentrating the SS from 0.16 to 5.6% DS content and with a positive impact in the subsequent MW drying process. The MW drying system removed *E. coli* below the detection limit and dehydrated the sludge up to a DS content of higher than 95%, at energy expenditures of 0.75 MJ kg^−1^ (0.2 kWh kg^−1^) and 5.5 MJ kg^−1^ (1.5 kWh kg^−1^), respectively. The energy expenditures of the MW drying system can be reduced by 10% and 30% by recovering the energy in the condensate and by combusting the dried sludge, respectively. The MW dried sludge, depending on crop sensitivity, is suitable for land application exhibiting a VS/DS ratio of below 0.6 and a DS concentration of higher than 90%. In addition, the dried sludge exhibited heavy metal concentrations below the land application standards (USEPA 1994). The combination of UF and RO filtration systems in series produced a high-quality permeate that is ideal for water reclamation in irrigation applications in accordance with Jordanian standard 893/2006. Due to the geographical location of Jordan, high solar irradiation was observed, which calls for PV systems to be applied for providing the evaluated system’s energy requirements.

## Data Availability

All data generated or analysed during this study are included in this published article.
